# Effect of Somatic Cell Count on Milk Production, Composition, Colour, Coagulation Properties and Cheese-Making Ability Traits in Manchega Dairy Sheep

**DOI:** 10.3390/foods15091527

**Published:** 2026-04-28

**Authors:** Imen Heddi, Javier Caballero-Villalobos, Nicolò Amalfitano, Fernando Martínez, Miguel Ángel Cantarero-Aparicio, Alessio Cecchinato, Manuel Ramón, Ana Garzón, Ramón Arias

**Affiliations:** 1 Centro Regional de Selección y Reproducción Animal de Castilla-La Mancha (CERSYRA), Instituto Regional de Investigación y Desarrollo Agroalimentario y Forestal de Castilla La Mancha (IRIAF), 13300 Valdepeñas, Spain; iheddi@jccm.es (I.H.); rarias@jccm.es (R.A.); 2 Departamento de Producción Animal, Universidad de Córdoba, 14071 Córdoba, Spain; t42caapm@uco.es (M.Á.C.-A.); pa1gasia@uco.es (A.G.); 3 Department of Agronomy, Food, Natural Resources, Animals and Environment (DAFNAE), University of Padova, Viale dell’Università 16, 35020 Padova, Italyalessio.cecchinato@unipd.it (A.C.); 4 Laboratorio Interprofesional Lácteo de Castilla-La Mancha (LILCAM), 45600 Talavera de la Reina, Spain; fmartinez@lilcam.org; 5 Department of Animal Breeding and Genetics, Instituto Nacional de Investigación y Tecnología Agraria y Alimentaria (INIA-CSIC), 28040 Madrid, Spain; manuel.ramon@inia.csic.es

**Keywords:** milk quality, somatic cell count, sheep, milk coagulation

## Abstract

Somatic cell count (SCC) in milk is widely used as an indicator of intramammary infections in dairy sheep and is routinely monitored by the dairy industry as a marker of milk quality. This study aimed to evaluate the effect of SCC levels on milk production, composition, colour, coagulation properties, and cheese-making ability in Manchega dairy sheep. A total of 752 individual milk samples were analysed. To normalise SCC distribution, the somatic cell score (SCS) was calculated and samples were classified into SCS classes. Increasing SCS significantly reduced daily milk yield and lactose content, increased milk pH, and decreased lightness (L*). Higher SCS was also associated with impaired coagulation properties, including longer rennet clotting time (RCT) and curd firming rate (k_20_), as well as reduced curd firmness (A_30_, A_60_). Similar effects were observed for modelled coagulation parameters, with delayed RCTeq and reduced kCF and CFp. Regarding cheese-making ability, SCS significantly affected curd humidity and protein recovery, whereas no significant effects were detected for dry curd yield or fat recovery. Overall, elevated somatic cell counts were associated with a reduction in the technological quality of Manchega sheep milk, particularly affecting coagulation behaviour and curd characteristics. These results underline the importance of controlling SCC levels in dairy sheep systems for both udder health monitoring and maintaining milk suitability for cheese-making.

## 1. Introduction

The production of sheep milk and cheese is highly important in the Mediterranean region, with traditional and high-quality products, many of which are protected by European Union quality schemes [1]. Spain is the second-largest producer of sheep milk in the EU, with 529,900 tonnes per year, and the third largest in the production of sheep’s milk cheese, averaging 72,160 tonnes per year [2].

The Manchega sheep breed, native to Spain, particularly in the La Mancha region, is well-known for its milk production quality. Enhancing milk production is one of the primary objectives of their breeding programme. Manchega sheep milk is highly prized for its high fat and protein content, making it the primary ingredient in the production of Manchego cheese, protected by a Denomination of Origin and highly esteemed worldwide. Approximately 60% of its production is exported [3].

Quality control of milk is one of the main objectives of the dairy sheep sector. Traditionally, this control was based on fat, protein, or dry matter content; later, hygienic–sanitary criteria such as total bacterial count and somatic cell count (SCC) have been incorporated. Total bacterial count reflects the hygienic quality of milk and is regulated under Regulation (EC) No. 853/2004 [4]. In contrast, SCC is widely used as an indicator of udder health and milk quality, as it is associated with mastitis (both clinical and subclinical) [5,6]. It is particularly useful for identifying subclinical cases, which do not present visible symptoms, although no specific threshold is established for sheep milk. Increased somatic cell count is an indicator of the onset of this inflammation and is associated with a loss of milk production [7] as well as changes in milk composition, including a decrease in lactose and casein, and an increase in proteolytic enzymes [8]. Given that there is a correlation between cheese quality and its physicochemical composition [9], these changes can affect the milk’s ability to coagulate properly during cheese and other dairy product manufacturing, leading to a significant drop in curd and cheese yields, as well as a less appealing texture in the final cheese [10].

Technological parameters of milk coagulation, such as rennet coagulation time, curd firming rate, and curd firmness, are commonly used to assess milk quality in terms of its behaviour during rennet coagulation [11,12]. These traits have been widely studied in dairy cattle [13] and in different breeds of dairy sheep [14,15], including their relationship with somatic cell count [16,17]. However, until now, there had not been studies conducted on the Manchega breed with modelled technological coagulation parameters, similar to other breeds such as Sarda [11,12], which are very interesting for studying other characteristics of the coagulation curve such as the asymptotic potential maximum value of curd firmness (CF_P_), the rennet coagulation time (RCT_eq_), the curd firmness instant rate constant (k_CF_), and the curd syneresis instant rate constant (k_SR_).

Similarly, parameters describing cheese-making ability are very interesting to verify the milk efficiency for cheese production. There are few studies available focused on cow [18] and sheep milk [19], but it would be interesting to conduct new research integrating parameters such as wet and dry curd yield, curd moisture, or recovery of fat and protein in the curd for the Manchega breed. These parameters are essential to evaluate cheese yield and processing efficiency, as wet curd yield reflects the total amount of curd obtained, whereas dry curd yield provides a more accurate estimation of the actual recovery of cheese solids, being less affected by water retention and therefore more representative of the real technological value of milk.

In this context, the main aim of the present study was to investigate the effect of different SCC levels in the milk of Manchega dairy sheep on (1) milk yield, composition, and colour traits; (2) traditional milk coagulation properties; (3) curd-firming model parameters (*CFt*); and (4) cheese-making ability traits.

## 2. Materials and Methods

### 2.1. Data Set and Collection of Milk Samples

Among animals with known genealogy (genetic traits of their parents), a total of 752 individual milk samples were randomly collected in 2022 from Manchega dairy sheep on five farms affiliated with the Spanish National Association of Manchega Sheep Breeders (AGRAMA) and located in the region of Castilla–La Mancha (Spain). Ewes were machine milked, and volumetric metres were used to determine daily milk yields (DMYs). Individual milk samples (250 mL) were collected from the morning milking and stored at 4 °C in labelled sterile airtight containers until analysis.

### 2.2. Laboratory Analysis

A first aliquot of 100 mL was analysed in the Experimental Lactology and Cheese Laboratory at the Regional Centre for Animal Selection and Reproduction (CERSYRA-IRIAF) (Valdepeñas, Ciudad Real, Spain). pH was measured using a Crison Basic20 pH metre (Crison Instruments S.A., Barcelona, Spain). Milk colour analysis was carried out using a Konica Minolta CM-2300d spectrophotometer (Konica Minolta Business Technologies, Inc., Osaka, Japan) [20]. Colour variables obtained included lightness (L*), redness (a*), and yellowness (b*).

Milk coagulation properties (MCP) were measured on a Formagraph type lactodinamograph (Ma.Pe. System, srl., Firenze, Italy) [21] with slight modifications. Briefly, 10 mL of milk was heated to 32°C, and 50 μL of a 4% single-strength liquid animal rennet dilution (70% chymosin and 30% pepsin, 185 international milk coagulating units/mL) was added. During the 60 min test, the equipment obtained values for: time (in minutes) between rennet addition and the start of milk coagulation (rennet coagulation time—RCT), the time (in minutes) necessary for the curd to reach a 20 mm firmness (curd-firming time—k_20_), and the curd firmness (in mm) measured at 30 and 60 min (A_30_ and A_60_, respectively).

Additionally, the modelled coagulation parameters were obtained using the following model [22]:

$$CFt={CF}_{P}\times \left\lfloor 1-e^{{-k}_{CF}\times (t-{RCT}_{eq})}\right\rfloor \times e^{{-k}_{SR}\times (t-{RCT}_{eq})}$$

This model uses all available information to derive the following parameters of curd firmness for each milk sample: CF_P_ (mm), representing the asymptotic potential maximum value of curd firmness at an infinite time; RCT_eq_ (min), indicating the rennet coagulation time estimated from equation; k_CF_ (%/min), representing the curd firmness instant rate constant; and k_SR_ (%/min), which is the curd syneresis instant rate constant.

A second aliquot of 50 mL was analysed in the Interprofessional Dairy Laboratory of Castilla–La Mancha (LILCAM), Talavera de la Reina, Spain. The percentage of fat (FAT), crude protein (CP), total solids (TS), casein (CAS), lactose (LAC), and the quantity of urea (U) (mg/L) were determined through mid-infrared spectroscopy on a Milkoscan 6000 FT (Foss Electric, Hillerød, Denmark). The casein index (CI) was calculated as CN/CP × 100. Somatic cell count (SCC) was performed on a Fossomatic FC (Foss Electric, Hillerød, Denmark). To normalise its distribution and stabilise variance, SCC was transformed into somatic cell score (SCS) using a log_2_ transformation:

$$SCS={log}_{2}\left(\frac{SCC}{10^{5}}\right)+3$$

This transformation was originally proposed to standardise SCC data and improve its statistical properties [23]. It is widely used in studies dealing with milk quality and technological properties, providing a biological interpretation, as each unit increase in SCS corresponds to a doubling of SCC [24,25]. According to their SCC, milk samples were divided into five different classes of equal range, which are fully described in Table 1.

A third aliquot was processed in the Dairy Small Ruminant Laboratory (Universidad de Córdoba, Córdoba, Spain) to determine curd yield. For this purpose, 40 mL of milk was heated in a thermostatic bath at 32 °C and 50 μL of a 4% single-strength liquid animal rennet dilution was added at the beginning of a 60 min analysis. Each resulting curd was collected in a numbered and weighed tube, ensuring complete identification and subsequently sliced with a spatula until it reached a grain size comparable to that obtained during the cutting of curd in cheese vats. Curd drainage was accelerated by centrifugation, a procedure commonly applied in laboratory-scale cheese yield determination to standardise whey expulsion and reduce experimental variability [26,27,28]. Accordingly, curds were centrifuged at 2800× *g* and 37 °C for 30 min, effectively separating the whey from the curd. This approach does not aim to reproduce industrial curd handling (e.g., cutting and stirring), but rather to provide a standardised and reproducible estimate of syneresis under laboratory conditions, allowing relative comparisons among samples. Drained curds were first weighed to obtain curd yield (**CY_curd_**) and then dried in an oven at 100 °C for 24 h to determine the dry curd solids (CY_solids_). Curd humidity (CH) was calculated as the difference between CY_curd_ and **CY_solids_**. Whey composition was finally obtained with a Milkoscan FT120 (Foss Electric, Hillerød, Denmark) to measure the recovery percentage of fat (REC_fat_) and protein (REC_prot_) in the fresh curd, using the following model [29]:

$${REC}_{fat}=\frac{{grams\;of\;fat}_{milk}-{grams\;of\;fat}_{whey}}{{grams\;of\;fat}_{milk}}$$

$${REC}_{prot}=\frac{{grams\;of\;protein}_{milk}-{grams\;of\;protein}_{whey}}{{grams\;of\;protein}_{milk}}$$

### 2.3. Statistical Analysis

The data set was checked for outliers using a threshold of ±3SD from the mean of each trait. To investigate the effect of the SCC (after log-transformation in SCS) on the aforementioned traits of milk yield, composition, and colour, traditional milk coagulation properties, curd-firming model parameters, and cheese-making ability parameters, the following linear mixed model was applied using the SAS MIXED procedure (SAS v.9.4, SAS Institute Inc., Cary, NC, USA):

*y_ijklm_* = *μ* + *DIM_i_* + *Parity_j_* + *Birth type_k_* + *SCS_l_* + *Flock-date_m_* + *e_ijklm_*

where *y_ijklm_* is the studied trait; μ is the overall intercept of the model; *DIM_i_* is the fixed effect of the *i*th class of days in milk (DIM), classified into three categories (≤90 days, 90–120 days, and >120 days); *Parity_j_* is the fixed effect of the *j*th class of parity order; *Birth type_k_* is the fixed effect of the *k*th class of parity type (2 classes: single or multiple lambing); *SCS_l_* is the fixed effect of the *l*th class of SCS; *Flock-date_m_* is the random effect of the *m*th flock-date record and is considered normally distributed ~N (0, $\sigma_{flock-date}^{2}$); and *e_ijklm_* is the residual random error and is considered normally distributed ~N (0, $\sigma_{e}^{2}$). The percentage of variance explained by flock date (flock date, %) was obtained for each trait by dividing the flock-date variance component ($\sigma_{flock-date}^{2}$) by the total random variance ($\sigma_{flock-date}^{2}+\sigma_{e}^{2}$).

To examine the response curve of the studied trait in function of the SCS class increase, polynomial contrasts (*p* < 0.05) were estimated; first-, second-, and third-order comparisons measured linear, quadratic, and cubic relationships, respectively.

## 3. Results

Descriptive statistics of single test-day milk yield, milk composition and colour, traditional coagulation properties, curd-firming time model parameters, and cheese-making ability traits are summarised in Table 2. Additionally, Table 3 shows results from linear mixed models based on different phenotypic factors (DIM, parity, birth type, SCS class, and flock date) applied to the aforementioned traits.

### 3.1. Milk Production and Composition

When comparing the coefficients of variation (CV), DMY and SCS showed the greatest range of variation (39.75 and 58.65, respectively). Among the other milk composition traits, FAT and UREA presented a CV of ~25%, followed by CAS, TS and CP, which ranged between 12 and 14%, while the lowest variability was measured in LAC and pH. As for milk colour indices, L* averaged 84.28, showing the lowest CV (0.62%), compared to a* and b*, which had a mean value of −1.62 and 7.79, respectively. The flock-date effect explained ~37% of DMY variation. Additionally, this trait was strongly affected by DIM. In terms of milk composition, flock date explained ~55% of variability for UREA, 45% for CI, between 25 and 31% for TS, FAT, LAC and pH, and around 18–22% for CP and CAS. DIM had a strong and significant effect on DMY and all composition traits. Moreover, Parity showed an effect on LAC, FAT and TS, whereas birth type only affected TS and FAT. Regarding colour variables, 12–14% of the observed variability for L* and a* was attributed to flock date, while DIM and parity strongly affected all colour traits. However, birth type only revealed a significant effect on L* and b*.

### 3.2. Milk Coagulation and Cheese-Making Traits

On average, both RCT and k_20_ were found to be widely variable, with CVs ranging from about 38 to 52%, highlighting a ~25 min difference between minimum and maximum RCT. It is also worth mentioning that approximately 2.6% of the samples did not coagulate under the described laboratory conditions. Differences were also evidenced for curd firmness, with a considerably higher variability for A_30_ (with a CV of ~54%), which was considerably lower for A_60_ (~18%). The flock-date factor explained 7–9% of the variability of RCT, k_20_ and A_60_, and around 11% for A_30_, suggesting a relatively lighter effect compared to that on both milk yield and compositional traits. Similarly, DIM and birth type only influenced A_60_, whereas parity did not exhibit significant effects on any MCP.

In terms of curd-firming modelled parameters (*CFt*), some relevant differences were found between these and traditional milk coagulation properties obtained on the lactodinamograph. RCT_eq_ was slightly faster than the traditional RCT. The k_CF_, representing the velocity of curd firming, showed an average of 10.43% per minute, with low variability. The k_SR_, representing syneresis, was 0.86% per minute, much lower than k_CF_ (also with low variability). Finally, CF_p_ (the potential maximum value of curd firmness) was found to be much higher than the traditional A_30_ trait. However, linear mixed models did show similar outcomes to those of traditional MCP. In this way, the variability explained by the flock date for RCT_eq_, k_CF,_ and CF_P_ was ~12%, and ~10% for k_SR_, while DIM showed a significant influence on k_CF_ and CF_p_. Contrastingly, parity and birth type only seemed to have a slight effect on CF_P_.

On the whole, cheese-making ability traits did not exhibit a large variability. It is only worth highlighting that curd humidity (considered as the main factor affecting CY) averaged 57.70%, and the recovery percentage of fat in the curd (REC_fat_) was found to be higher than that of protein (REC_protein_): 94.41 vs. 80.81%. Variance explained by the flock date was found to be higher than that of both MCP and *CFt* traits, as this effect is responsible for 18–30% of the observed variability for all cheese-making ability traits. Cheese-making ability was also strongly affected by DIM, although the effect on REC_protein_ was somewhat milder. Moreover, parity affected curd yields and humidity, while birth type had a slight impact on solids, CH and the recovery of protein and fat in the curd.

### 3.3. Effect of SCS on Milk Composition and Colour

**Figure 1:**
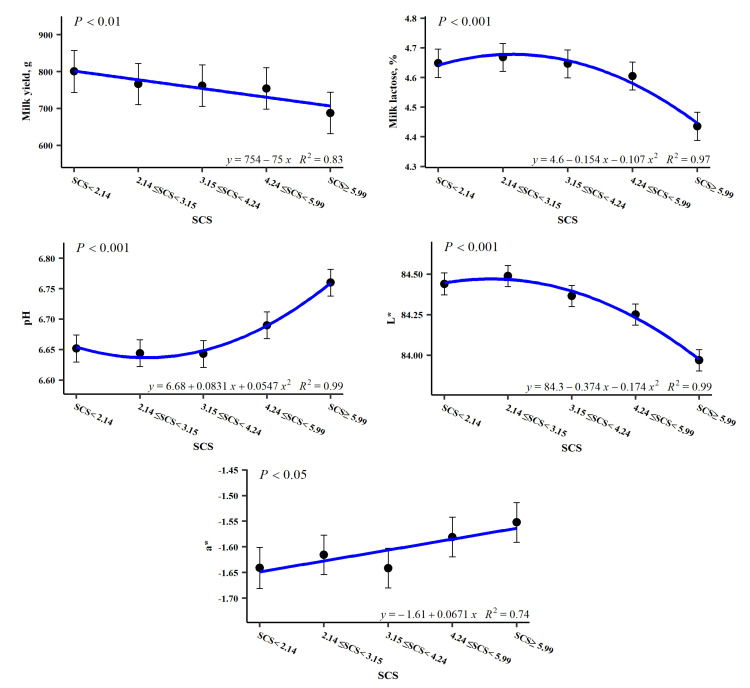
Least square means for the significant (at least *p* < 0.05) effect of the somatic cell score (SCS) on the traits of milk production, composition, and colour.

The average SCS (log_2_ cells/mL) for this study was 4.06 (equivalent to SCC = 208,300 cells/mL) with a high standard deviation and variability (Table 2). SCS strongly influenced DMY, LAC, pH and L*. A minor effect was found on b*, while there were no significant effects on TS, FAT, UREA or protein-related variables. Figure 1 illustrates the least square means (LSMs) for the significant impact of SCS on the investigated traits, along with the corresponding curve of the data across the different SCS classes (according to the significant linear or quadratic contrasts obtained).

DMY displayed a significant linear pattern, with a drop in milk production with increasing SCS. The colour trait a* also exhibits a linear pattern, but opposite to that of DMY, increasing with SCS. LAC shows a clear quadratic pattern, being lower for the first SCC class (SCS < 2.14 log_2_ cells/mL), increasing in the second class (SCS = 2.14–3.15 log_2_ cells/mL), and decreasing as somatic cells rise, especially when SCS > 5.99 log_2_ cells/mL. This pattern was also found to be similar for lightness (L*). Meanwhile, pH also exhibits a quadratic pattern, but as a mirror image of the two previous traits; that is, it has a higher value for the first SCC class, decreases in the second, and increases almost linearly as the SCS of the milk increases. Contrastingly, FAT, CP, CAS, and UREA, as well as the yellowness index (b*), were not found to be affected by SCS.

### 3.4. Effect of SCS on Milk Coagulation and Cheese-Making Ability

**Figure 2:**
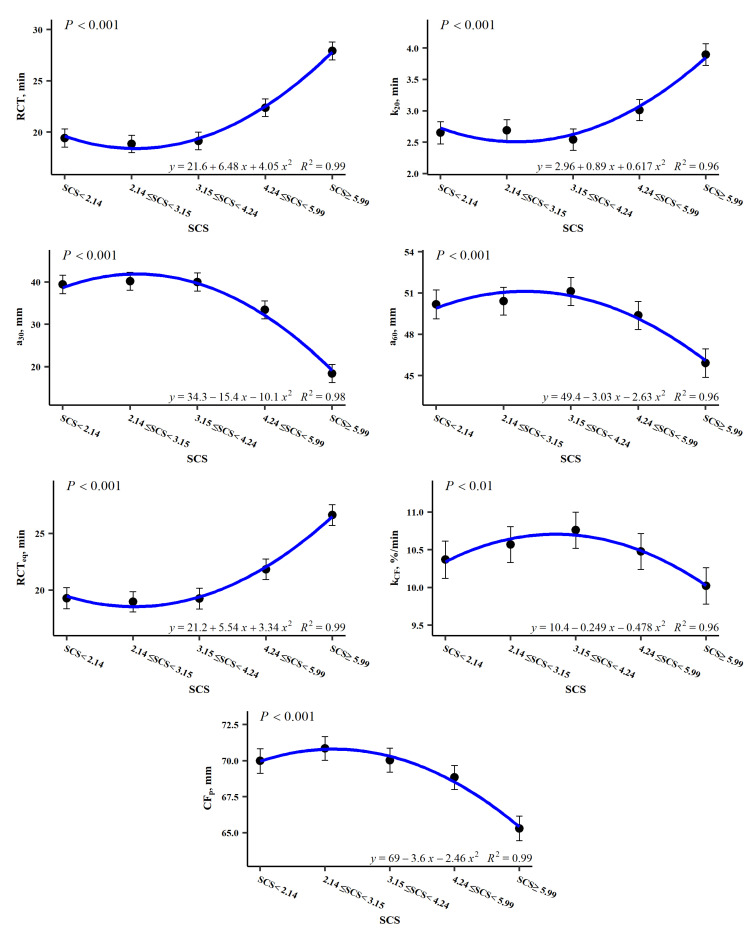
Least square means for the significant (at least *p* < 0.05) effect of the somatic cell score (SCS) on the traditional milk coagulation properties (MCP).

The LSM for the SCS effects on traditional milk coagulation properties is presented in Figure 2. RCT showed a positive quadratic pattern with very large differences depending on SCS, being larger for the first class (SCS < 2.14 log_2_ cells/mL), decreasing in the second class (2.14 < SCS < 3.15 log_2_ cells/mL), and increasing considerably as the SCS increases, especially in the last class (SCS > 5.99 log_2_ cells/mL). Regarding curd firming time, k_20_ exhibits a quadratic pattern similar to that of RCT, although with less pronounced differences. Curd firmness parameters follow a pattern opposite to the previous MCP traits. That is, A_30_ and A_60_ are lower in the first class (SCS < 2.14 log_2_ cells/mL), slightly increasing in the second class (2.14 < SCS < 3.15 log_2_ cells/mL), and progressively decreasing with higher SCS, especially in the last class (SCS > 5.99 log_2_ cells/mL), with differences being more pronounced for A_30_ than for A_60_.

**Figure 3:**
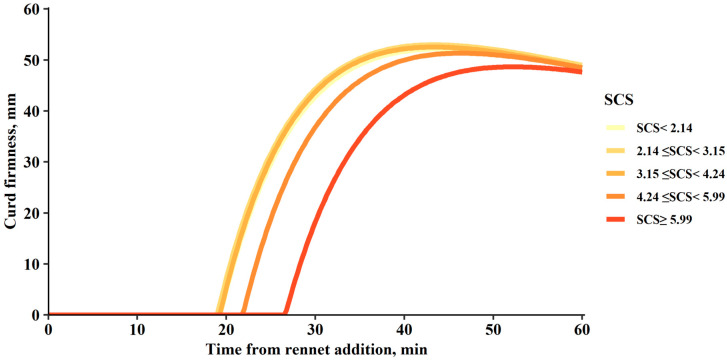
Pattern of curd firmness after rennet addition (*CFt* modelling) of milk samples according to the somatic cell score (SCS). The classes of the SCS effect correspond to the SCS categories defined in Table 1, based on predefined ranges of somatic cell score.

Regarding curd-firming modelled parameters, RCT_eq_ and CF_P_ were strongly influenced by SCS. A slight effect of somatic cells on k_CF_ was also evidenced, while no impact was observed on k_SR_ (Table 3). Figure 3 shows the patterns of *CFt* modelling parameters according to SCS levels. As illustrated in this figure, the five classes of SCS can be categorised into three major groups. A low-SCS group (<4.24 log_2_ cells/mL), a mid-SCS group with a SCS range from 4.24 to 5.99 log_2_ cells/mL, and a high-SCS group (>5.99 log_2_ cells/mL). The low-SCS group, which included the first three classes of SCS, initiated coagulation approximately 20 min after rennet addition, and showed a high asymptotic CF_P_. From this point, coagulation time increased with cell count, while firmness decreased, with both patterns being especially evident when SCS > 5.99 log_2_ cells/mL. In addition, syneresis was found to be more pronounced in classes with higher curd firmness.

**Figure 4:**
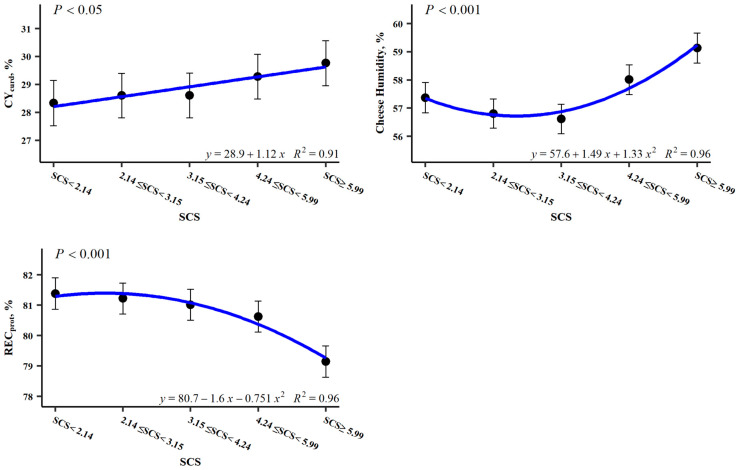
Least square means for the significant (at least *p* < 0.05) effect of the somatic cell score (SCS) on the traits of cheese-making efficiency.

As far as cheese-making ability is concerned, SCS significantly affected CH and REC_protein_ and, to a lesser extent, CY_curd_ (Table 3). This effect of SCS levels on cheese-making ability traits is represented in Figure 4. CY_curd_ shows a linear pattern, with a very small increase in yield when cell counts increase, which may be related to the quadratic pattern of curd humidity. CH is slightly higher in the first cell count class (SCS < 2.14 log_2_ cells/mL) than in the next two classes, with an evident increase in moisture over the threshold of 4.24 log_2_ cells/mL. Meanwhile, REC_protein_ showed a quadratic pattern, with a slight decrease in the first SCS classes, which becomes more evident with high cell counts (SCS > 5.99 log_2_ cells/mL). However, no differences were found for REC_fat_.

## 4. Discussion

### 4.1. Composition and Colour of Milk

In the Mediterranean basin, native sheep breeds are strongly linked to the tradition and development of rural areas, leading to the manufacture of high-standard agri-food products, often labelled under quality marks such as Protected Designation of Origin (PDO) and Protected Geographical Indication (PGI) cheeses [1,30]. Conservation and genetic selection of these small ruminant populations acquire great importance, since many studies have proved that the composition of milk from autochthonous breeds (mainly due to its higher fat and protein content) is more favourable for cheese-making compared to other foreign third-country breeds [31,32]. Hence, the importance of understanding in detail the aspects that can affect the composition and coagulation of milk, so as to improve quality, sustainability and profitability of a sector so deeply rooted in the culture of southern European areas.

Considering the aforementioned facts, milk composition in the present study is in line with that previously reported in the literature, both for Manchega and other native Spanish [33,34,35], Italian [36,37] and French breeds [38,39]. It is worth highlighting the significant proportion of casein compared to total protein, revealing a substantial amount of coagulable casein, thus evidencing a remarkable cheese-making potential of Manchega sheep milk, exhibiting levels similar to other native breeds such as Comisana, and even higher than those of Sarda [40,41]. This trait is crucial for cheese production and strongly affects coagulation and the rheological characteristics of the gel, also exhibiting, along with lactose concentration, the least variability of all compositional parameters. Therefore, assessment of the coagulable protein is deemed important, so as not to overestimate the total milk protein, which also contains whey proteins that are not directly involved in the coagulation process.

The assessment of milk colour represents a rapid and informative approach for evaluating milk quality, as colour indices are associated with both compositional and technological properties of milk. Previous studies in Manchega sheep milk have reported significant relationships between colour parameters and milk composition. In particular, lightness (L*) has been negatively correlated with protein, total solids, and casein content, and positively correlated with lactose [42]. Similarly, redness (a*) and yellowness (b*) indices have been positively associated with fat, protein, and total solids, and negatively related to lactose content. In addition, colour values have shown strong correlations with key compositional traits such as fat, protein, and total solids, and can contribute to predicting milk coagulation behaviour and technological quality [34]. This relationship is supported by multivariate approaches showing that colour parameters are integrated within the overall structure of milk quality traits, being linked to composition, coagulation, and curd yield [14]. Moreover, milk colour is influenced by physicochemical factors such as pH and urea, as well as by dietary components, particularly carotenoids, riboflavin, and fat content, which affect light scattering and pigmentation. Therefore, colour parameters can provide indirect information on milk composition, nutritional status, and technological behaviour, supporting their use as practical indicators of milk quality.

In addition to colour-related traits, the proportion of variability explained by the flock-date factor differed among milk composition traits, being particularly high for urea content and casein index (CI). This finding highlights the relevance of evaluating the protein profile and nitrogen-related compounds in sheep milk, given their strong relationship with feeding conditions [43]. In addition, days in milk (DIM) showed a substantial effect on most compositional traits, with the exception of pH, in agreement with previous studies [37,44]. A similarly pronounced influence of DIM was also observed for milk colour, exceeding that of other factors included in the model, such as parity and birth type.

Regarding technological quality, the average MCP traits reveal a curve similar to that described for other Spanish breeds, but different from other breeds like Sarda. For instance, one study describes the typical pattern for Sarda sheep milk [41]: it coagulates quickly (8 min), showing a high firmness at 30 min (A_30_ = 48 mm), which decreases at 60 min (A_60_ = 40 mm), and a pattern was also observed by other authors for the same breed [11]. In contrast, our results suggest that Manchega milk takes longer to coagulate (RCT = 21.36 min), showing similar k_20_ and curd firmness that progressively increases (A_30_ = 34.58 mm, A_60_ = 49.48 mm), as previously described [34]. Another notable difference is the proportion of non-coagulating milk samples (2.26%), which was found to be higher than what other authors reported for sheep milk (~0.4%) [15] or cow milk (~4%) [45] in similar laboratory conditions. These differences are attributed to multiple factors, mainly milk composition, as already pointed out both in cattle [46] and dairy sheep [47]. Likewise, differences may also be due to genetic factors linked to the breed itself, such as casein profiles [48], and even to differences in the experimental conditions of each analysis in relation to temperature, initial pH of milk, or quality and composition of rennet [49]. However, the well-known limitations of the lactodinamograph regarding classic MCP parameters [15,50] do not appear to be the cause, since the modelled parameters (*CFt*) seem to behave in a similar way to traditional MCP, except for the syneresis indicator, which in our study was found to be similar to that reported for other breeds [22]. Furthermore, with the exception of coagulation time, the present study revealed less variation (CV) for the modelled parameters (*CFt*) than for traditional MCP, which suggests that these parameters better describe the coagulation curve.

As for animal-related fixed and random effects, the variability of the flock-date factor (%) was found to be moderate for coagulation parameters. It is worth highlighting the significant effect of DIM only for A_60_, k_CF_, and CF_p_, which could be linked to the content and stability of caseins that determine the structure of the curd. This would also be supported by the effect of parity and birth type on CF_p_, which suggests that casein characteristics may influence coagulation properties of Manchega milk to a greater extent than other traits such as pH or rennet activity, as also proposed by other studies [45].

Furthermore, milk from Manchega stands out for its good curd yield and solids recovery, which may be related to the aforementioned compositional traits [51]. The reported curd yield (28.7%) is in alignment with previous studies from our research group for the same breed (~26.8%) [14]. However, when compared to other Spanish sheep breeds, CY was found to be lower than average values for Merino from Valle de Los Pedroches (~38%) and Merino de Grazalema (~31.3%), yet higher than for the highly productive Assaf (CY = 25.2%) [35].

Comparisons with studies using different cheese-making methods are more challenging; nevertheless, the curd recovery percentages obtained in the present study show a trend similar to that reported for Sarda sheep (REC_PROTEIN_ = 76.7% and REC_FAT_ = 94.0%) [52], with higher recovery values for fat than for protein. This pattern has been previously described when the fat-to-protein ratio in milk is high [53]. It should also be noted that the flock-date factor had a greater effect on these parameters than on the experimental coagulation traits (MCP and *CFt*). Previous studies on sheep milk have shown that short-term variations in management, feeding, microenvironment, and seasonality can affect curd yield and moisture by influencing milk composition and the proportion of protein and fat recovered in the curd [37,44]. Consistent with our results, these studies also report an effect of DIM and parity on cheese-making traits, including a relevant impact on curd yield and protein recovery, likely reflecting changes in milk production and composition throughout lactation.

### 4.2. SCS Effects on Sheep Milk

Somatic cell count is considered a good indicator for the detection of mastitis (both clinical and subclinical), a major concern for dairy farms due to the significant economic losses it causes in terms of animal health, milk production, and quality [54,55]. It is particularly useful for identifying subclinical mastitis, which does not present visible symptoms. The average SCS in the present study (4.06 log_2_ cells/mL) displayed a high variability that can be explained by the different cellular responses of the various causative microorganisms, and also by other non-infectious factors that acquire particular relevance in dairy small ruminants [56]. In any case, the average SCC reported in this study is very close to the threshold established at 200,000 cells/mL for the diagnosis of subclinical mastitis in Manchega sheep [57], which corresponds to approximately 4.0 on the SCS scale. These values are also consistent with those described for Manchega [58] and Sarda sheep [52].

#### 4.2.1. Effects on Milk Yield and Composition

The present study shows a progressive drop in milk production (up to 11.38%) associated with increasing somatic cell count, consistent with the well-known impact of mastitis on milk yield. Similar effects have been widely reported in both small ruminants [8,16,56,59,60,61] and dairy cattle [62]. This relationship has also been reported in dairy sheep populations and has persisted in recent years despite the routine implementation of mastitis prevention and control programmes in dairy sheep farms. The effectiveness of these programmes has been further constrained by the restrictions on the use of intramammary antibiotics introduced by Regulation (EU) 2019/6 [63]. This situation occurs in a context of increasing milk production in sheep enrolled in the Manchega Breeding Programme and highly efficient management practices in increasingly technologically advanced farms [64]. In response, the breeding programme has incorporated improved mammary morphology as a genetic selection criterion to facilitate milk storage and milkability and thus reduce the risk of mastitis associated with these factors [65]. Similarly, the present study also described a decrease in lactose content associated with somatic cell count, a relationship previously reported in milk from small ruminants [10,66,67] and dairy cows [68,69]. This effect has been attributed to damage to mammary epithelial cells, which may impair lactose synthesis [54,68]. Furthermore, epithelial tissue damage caused by mastitis can increase the permeability of the mammary gland, allowing lactose to pass from milk into the bloodstream and promoting the transfer of mineral components (such as citrates, bicarbonates, sodium, and chloride ions) from blood serum into milk. These changes contribute to an increase in milk pH [10,12,70,71], a pattern also observed in our results.

Regarding colourimetric parameters, references in the literature are scarce. In the present study, lightness (L*) diminished as somatic cell count increased, showing a trend similar to that observed for DMY and LAC. This effect could be related to changes in light scattering in milk with higher cell content, as well as to mineral and micellar alterations [56,72]. In addition, a slight increase in the redness index (a*) was observed with increasing SCS, a phenomenon previously described in cow milk [73,74]. This reddish colouration may be associated with the presence of blood components, such as haemoglobin, linked to mammary tissue damage during mastitis [75]. Moreover, milk colour can also be influenced by dietary factors, particularly the content of carotenoids and retinoids, which may contribute to variations in colour indices. These results support the potential use of colour indices as tools for milk quality control, particularly considering that previous studies from our group have also demonstrated their usefulness for predicting milk composition and coagulation behaviour [76].

Furthermore, somatic cell count did not significantly affect the main milk components (fat and protein). Previous studies have reported conflicting results regarding the relationship between somatic cell count and milk fat content. Some authors describe a decrease in fat content associated with increased cell count, attributed to udder damage, impaired fat synthesis in the mammary gland, or increased lipase activity [56,77,78], whereas others report an increase, explained by a concentration effect resulting from reduced milk yield [79]. Moreover, large-scale field studies indicate that the effect of somatic cell count on milk composition can vary depending on farm conditions, animal health status, and management practices [80]. Other studies, however, report no significant change in fat percentage with increasing somatic cell count [81,82]. Several explanations have been proposed for the absence of changes in fat percentage. One relates to the degree of inflammation caused by subclinical mastitis: when inflammation is mild, it may not cause substantial damage to mammary epithelial cells or significantly impair their capacity for fat synthesis [81]. In breeds characterised by relatively high milk fat content, such as Manchega, the effect of somatic cell count on fat percentage may therefore be less pronounced. Moreover, if fat yield and milk yield decrease proportionally, fat percentage may remain stable despite a reduction in total fat content in the milk [81,83].

Similar inconsistencies have been reported regarding the effect of somatic cells on protein and, particularly, casein content. Some studies report an increase in protein concentration, attributed to the extravasation of serum proteins as a consequence of inflammation [17,78], whereas others report a decrease associated with reduced synthetic activity of the mammary gland or with the action of proteolytic enzymes, such as plasmin, as well as enzymes derived from somatic cells (e.g., cathepsin D and elastase), linked to elevated somatic cell counts [66,77,79,84,85,86]. Conversely, several studies, including the present one, have reported no significant changes in total protein or casein content [59,83]. Nevertheless, alterations in the micellar structure of casein have been described even in the absence of changes in total casein concentration, which may affect the coagulation properties of milk [87]. Therefore, further investigation of these protein fractions and their stability is required to better understand their role in determining milk suitability for coagulation, particularly given the still-limited scientific evidence available.

#### 4.2.2. Effects on Milk Coagulation

The results of the present study support the widely accepted relationship between high somatic cell counts in milk and impaired coagulation properties. Previous studies evaluating traditional MCP have shown that increasing SCC is associated with longer coagulation time (RCT) and curd firming time (k_20_), together with reduced curd firmness (A_30_ and A_60_) [16,17,83]. A similar pattern was observed in our results, particularly in the two highest cell count classes (SCS ≥ 4.24 log_2_ cells/mL).

It is also important to highlight the magnitude of the differences observed in MCP across somatic cell score classes. For RCT, an increase of up to 8.49 min was observed between the second and fifth SCS classes, corresponding to a 30.41% increase, which is slightly lower than the 5 min difference reported between extreme SCC levels by another study [88]. Similarly, some authors reported that RCT can double in milk from ewes affected by mastitis compared with milk from healthy animals [89]. An increase of up to 31.96% in k_20_ was also observed between these classes. Regarding curd firmness, A_30_ decreased markedly, with a reduction of up to 117%, whereas the decrease in A_60_ was much smaller (9.31%). These results suggest that somatic cells affect milk coagulation parameters to different extents depending on the specific trait considered. An additional point worth noting is the somewhat paradoxical pattern observed between the first and second SCS classes: RCT and k_20_ increased, while A_30_ and A_60_ decreased (Figure 2), deviating from the general trend observed across the remaining classes. A similar behaviour for RCT has also been reported in dairy cows [68]. One possible explanation proposed in previous studies is that extremely low SCC levels may be linked to host-related factors affecting the immune response to intramammary infections [90,91]. However, this interpretation should be considered with caution in the context of the present study, as no direct immunological measurements were performed. In healthy animals, milk normally contains a resident population of leukocytes that plays an important role in the innate immune defence of the mammary gland. Therefore, extremely low SCC values could reflect animals with a limited immune response capacity, potentially allowing subclinical or undetected mastitis processes to occur. Such conditions could also lead to subtle changes in milk production, lactose content, or milk pH, as suggested by the patterns observed in Figure 1. As discussed previously with regard to the definition of appropriate SCS thresholds, further research is needed to clarify whether animals with very low SCC may exhibit reduced immune responsiveness. This issue may be particularly relevant in dairy sheep, especially if SCC is to be used as an indicator trait in genetic selection programmes aimed at improving mastitis resistance. In line with this hypothesis, some authors reported a strong genetic correlation between non-coagulating milk and somatic cell count, suggesting that the *loci* associated with impaired milk coagulation and increased SCC are closely linked or partially overlapping [92].

It is widely accepted that ewes with high somatic cell counts, typically associated with mastitis (particularly subclinical cases), experience inflammation of the mammary gland. Although no universal threshold is established for sheep milk, values above approximately 500,000 cells/mL are commonly associated with an increased likelihood of intramammary infection; however, this may also be influenced by non-infectious factors such as stage of lactation, parity, breed, and overall flock health status. This condition alters the permeability of the mammary epithelium and the mineral balance of milk, allowing proteolytic enzymes from the bloodstream to enter the mammary secretion and cleave caseins [10,17,70]. It should also be noted that total bacterial count is an important hygienic–sanitary parameter in milk quality assessment; however, the present discussion focuses specifically on SCC as an indicator of udder health and its relationship with milk technological properties. Although these changes may not necessarily affect the total casein percentage, they can modify the micellar structure and the distribution of casein fractions, leading to delayed coagulation and weaker curds [56]. In the present study, the increase in somatic cell count was also associated with reduced lactose content and higher milk pH, factors that may further impair coagulation by altering the acidification process and delaying casein demineralisation, thereby reducing the effectiveness of rennet enzymes [12,49,71]. Therefore, further investigation of casein micellar structure and casein fraction profiles in relation to somatic cell count would be of particular interest in dairy sheep.

Regarding the modelled curd firmness parameters, Figure 3 shows that these were also affected by SCS, further confirming the detrimental effect of mastitis on milk coagulation. RCT_eq_ was influenced by SCS in a pattern similar to that observed for RCT, with coagulation time increasing by up to 28.73% as cell count increased, while k_CF_ decreased by up to 5.47% at the highest SCS levels. Although studies evaluating the effect of SCS on CFt parameters are limited, some evidence is available from research conducted in dairy cows. In agreement with our results, a previous study reported a significant effect of SCS on both RCT_eq_ and k_CF_, observing delayed coagulation and reduced gel formation rate as cell count increased [68]. Similar findings were also described by other authors, who related these effects partly to changes in milk pH [41]. The effect of SCS on CF_p_ was also evident, with reductions of up to 5.47% at higher cell counts, supporting a similar interpretation to that observed for A_60_. The coagulation patterns shown in Figure 4, consistent with the results presented in Figure 2, evidence that milk with an SCS ≥ 4.24 log_2_ cells/mL (SCC = 239,199 cells/mL) exhibits impaired coagulation, characterised by a delayed onset of gelation and reduced potential curd firmness, a pattern also reported in Sarda sheep [12]. Previous studies conducted in Manchega sheep and other Spanish breeds under comparable management conditions have proposed somatic cell count thresholds between 250,000 and 300,000 cells/mL to discriminate animals with subclinical mastitis [57,93], values that are very close to those observed in the present study. Although defining a precise threshold for milk suitability for coagulation based solely on SCC may be uncertain, the results obtained here show a clear parallel with studies that use SCC as an indirect indicator of mastitis. As mentioned earlier, these findings highlight the need for further research in Manchega sheep, particularly regarding the infectious, immunological, and genetic factors involved.

#### 4.2.3. Effects on Cheese-Making Efficiency

When somatic cell count increased, the slight rise in curd yield (CY_curd_; up to 4.82%) appeared to be associated with a concomitant increase in curd moisture (up to 3.93%). This relationship was supported by the strong correlation observed between both variables in the present study (r = 0.761, *p* < 0.01; unpublished data). Previous studies have also reported increased moisture with increasing somatic cell count or score (SCS) [17,94,95,96], which may bias the estimation of curd yield [97]. This interpretation is consistent with the variability reported in the literature. Some studies have found no effect of SCS on curd yield, including those conducted in sheep [52,83] and in bovine milk [94]. In contrast, other studies have reported a negative effect of somatic cells on rennet coagulation properties and cheese yield, particularly in Parmigiano Reggiano and other cow milk cheeses [68,98]. It should be noted that somatic cells are naturally present in milk [56], and their effect on milk properties depends on their concentration. In sheep milk, values below approximately 200,000 cells/mL are generally associated with healthy udders [99,100], whereas levels above 400,000 cells/mL are more indicative of intramammary infection [101], with intermediate ranges showing variable effects.

In the present study, we also observed substantial variability attributable to the flock–date effect for cheese-making aptitude traits. This variability likely reflects differences in milk composition and environmental conditions, including alterations in protein fractions [96,98] and changes in the physicochemical properties of casein micelles. Such changes may impair curd aggregation and syneresis, ultimately contributing to increased moisture retention in the curd [77]. These patterns were also reflected in the decrease in protein recovery (REC_protein_) observed with increasing SCS, consistent with previous reports in both ovine and bovine milk [68,83]. In contrast, other authors reported no significant differences in REC_protein_, although they observed a marked effect on fat recovery (REC_fat_) [77]. However, no effect of SCS on REC_fat_ was detected in the present study. In addition, increased milk pH associated with elevated somatic cell counts may impair syneresis, leading to higher curd moisture and, consequently, lower curd yields, as well as reduced recovery of fat and protein in cheese [95]. Overall, these findings highlight the considerable variability in milk cheese-making aptitude and, in particular, in the response to somatic cell count.

Further research is therefore warranted to better elucidate the mechanisms underlying these effects, especially regarding factors influencing coagulation in sheep milk. These include pH, as well as changes in casein fractions, their structural integrity during curd formation, their influence on curd moisture, and their capacity to retain fat. These aspects are critical not only for cheese yield but also for ensuring proper cheese ripening.

## 5. Conclusions

This study shows that somatic cell score (SCS) significantly affects the technological quality of Manchega sheep milk, with clear implications for cheese production. Increasing SCS was associated with reduced milk yield and lactose content, higher milk pH, and changes in colour, whereas fat and protein concentrations remained largely unaffected. Despite this, elevated SCS markedly impaired milk coagulation, leading to delayed coagulation and reduced curd firmness, as consistently shown by both traditional and modelled parameters. The effect on cheese-making performance was mainly related to changes in curd properties rather than yield, as higher SCS increased curd moisture and reduced protein recovery, potentially biasing yield estimation. Overall, these results indicate that somatic cells primarily affect milk technological functionality and curd structure rather than gross composition, ultimately compromising both milk suitability for cheese production and the quality of the final cheese. The observed patterns suggest the existence of practical SCS thresholds affecting milk technological performance, although further research is required to define them accurately. Therefore, controlling SCS is essential not only for udder health but also for maintaining optimal processing performance, and coagulation traits may serve as useful complementary indicators of milk technological quality.

## Figures and Tables

**Table 1:** Somatic cell classes and ranges (SCC ranges were derived from SCS classes based on a log_2_ transformation and are presented as rounded values for clarity).

Somatic Cell Class	Range (SCS, log_2_ Scale)	SCC (Cells/mL)	Animals (*n*)
SCS_1_	<2.14	<55,000	146
SCS_2_	2.14–3.15	55,000–110,000	158
SCS_3_	3.15–4.24	110,000–235,000	149
SCS_4_	4.24–5.99	235,000–795,000	149
SCS_5_	>5.99	>795,000	150

**Table 2:** Descriptive statistics of single test-day milk yield (DMY), milk composition, milk colour, traditional coagulation properties, curd-firming time (*CFt*) modelled parameters, and cheese-making ability traits (n = 752).

Trait ^1^	Mean	SD	P5	P95	CV
DMY, g	755.76	300.42	350.00	1300.00	39.75
Milk composition					
TS, %	19.03	2.41	15.05	23.19	12.69
FAT, %	7.57	1.90	4.45	10.69	25.07
CP, %	5.83	0.72	4.68	7.06	12.30
CAS, %	4.70	0.66	3.62	5.84	13.99
CI	0.80	0.02	0.77	0.84	2.95
LAC, %	4.62	0.34	3.99	5.11	7.28
UREA, mg/L	498.55	124.68	280.00	692.00	25.01
SCS	4.06	2.38	0.94	8.91	58.65
pH	6.68	0.13	6.48	6.90	1.97
Milk colour					
L*	84.28	0.52	83.37	85.06	0.62
a*	−1.62	0.34	−2.23	−1.11	−20.74
b*	7.79	0.94	6.14	9.37	12.05
Traditional coagulation properties					
RCT, min	21.36	8.03	11.12	37.62	37.61
k_20_, min	2.94	1.52	1.50	6.00	51.86
A_30_, mm	34.58	18.55	0.00	56.84	53.64
A_60_, mm	49.48	8.79	32.34	61.74	17.77
*CF_t_* parameters					
RCT_eq_, min	20.85	7.39	11.13	35.69	35.43
k_CF_, %/min	10.43	1.84	7.70	14.12	17.64
k_SR_, %/min	0.86	0.15	0.70	1.16	16.95
CF_p_, mm	69.03	6.69	55.15	77.48	9.70
Cheese-making ability					
CY_curd_, %	28.70	5.23	20.60	37.70	18.22
CY_solids_, %	12.26	2.38	8.40	16.50	19.43
CH, %	57.70	3.58	52.15	63.84	6.20
REC_protein_, %	80.81	2.86	76.40	84.95	3.53
REC_fat_, %	94.41	2.30	90.57	97.60	2.44

P5 = 5th percentile; P95 = 95th percentile. ^1^ DMY= daily milk yield; TSs = total solids; FAT = fat content; CP = crude protein; CAS = casein; CI = casein index; LAC = lactose; SCS = log_2_ (SCC/100,000) + 3; RCT = rennet coagulation time; RCT_eq_ = estimated RCT; k_20_ = time from coagulation to a curd firmness of 20 mm; A_30_ = curd firmness 30 min after rennet addition; A_60_ = curd firmness 60 min after rennet addition; CF_p_ = asymptotical potential value of curd firmness; k_CF_ = curd-firming instant rate constant; k_SR_ = syneresis instant rate constant; CF_max_ = maximum curd firmness achieved within 90 min; CY_curd_ = cheese yield in terms of fresh curd; CY_solids_ = cheese yield in terms of solids retained in the curd; CH = curd humidity; REC_protein_ = recovery of protein from the milk into the fresh curd; REC_fat_ = recovery of fat from the milk into the fresh curd.

**Table 3:** Results from linear mixed models based on days in milk (DIM), parity order, birth type (single or multiple lambing), and SCS class (20% of animals per class) applied to single test-day milk yield (dMY), milk composition, milk colour, traditional coagulation properties (MCPs), curd-firming model parameters (CFt), and cheese-making ability traits.

Trait ^1^	Fixed				Flock Date,% ^2^	RMSE ^3^
	DIM	Parity	Birth Type	SCS		
DMY, g	14.06 ***	1.92	3.4	3.91 **	36.79	228.96
Milk composition						
TS, %	76.41 ***	7.16 ***	5.66 *	0.76	26.84	1.67
FAT, %	53.01 ***	8.67 ***	6.83 **	1.59	31.50	1.32
CP, %	82.36 ***	1.67	0.15	1.15	18.33	0.53
CAS, %	90.5 ***	2.69 *	0.57	1.28	22.67	0.47
CI	51.24 ***	4.93 ***	8.13 **	2.17	45.57	0.02
LAC, %	51.75 ***	6.45 ***	1.84	19.56 ***	24.57	0.25
UREA, mg/L	27.07 ***	1.47	1.06	1.22	55.23	75.61
pH	6.16 **	1.39	0.01	29.74 ***	29.07	0.10
Milk colour						
L*	33.6 ***	4.97 ***	4.34 *	28.05 ***	14.08	0.44
a*	43.02 ***	4.36 ***	1.17	2.54 *	12.04	0.27
b*	17.63 ***	5.9 ***	3.97 *	1.19	12.76	0.84
Traditional coagulation properties						
RCT, min	0.75	1.03	2.43	36.57 ***	7.33	7.09
k_20_, min	1.48	1.63	1.98	19.97 ***	7.63	1.39
A_30_, mm	0.41	0.86	0.91	46.24 ***	10.97	15.74
A_60_, mm	6.83 **	0.67	5.65 *	8.4 ***	8.80	8.05
*CF_t_* parameters						
RCT_eq_, min	0.34	2.03	2.07	28.86 ***	11.38	6.54
k_CF_, %/min	4.97 **	0.98	1.77	3.6 **	12.38	1.68
k_SR_, %/min	1.75	2.08	1.62	1.32	9.92	0.14
CF_p_, mm	7.29 ***	2.49 *	5.2 *	16.89 ***	12.29	5.87
Cheese-making ability						
CY_curd_, %	35.7 ***	3.49 **	2.96	2.41 *	26.45	3.96
CY_solids_, %	64.41 ***	4.41 ***	5.48 *	0.57	26.06	1.69
CH, %	17.08 ***	4.83 ***	4.46 *	12.79 ***	18.64	3.09
REC_protein_, %	3.84 *	1.03	5.39 *	14.15 ***	29.07	2.39
REC_fat_, %	9.84 ***	1.35	6.62 *	0.86	22.83	2.06

* *p* < 0.05; ** *p* < 0.01; *** *p* < 0.001. ^1^ DMY= daily milk yield; TSs = total solids; CP = crude protein; CAS = casein; CI = casein index; LAC = lactose; SCS = log_2_ (SCC/100,000) + 3; RCT = rennet coagulation time; RCTeq = estimated RCT; k_20_ = time from coagulation to a curd firmness of 20 mm; A_30_ = curd firmness 30 min after rennet addition; A_60_ = curd firmness 60 min after rennet addition; CF_p_ = asymptotical potential value of curd firmness; k_CF_ = curd-firming instant rate constant; k_SR_ = syneresis instant rate constant; CY_curd_ = cheese yield in terms of fresh curd; CY_solids_ = cheese yield in terms of solids retained in the curd; CH = curd humidity; REC_protein_ = recovery of protein from the milk into the fresh curd; REC_fat_ = recovery of fat from the milk into the fresh curd. ^2^ Flock date, % = percentage of variance explained by the flock-date random effect on the sum of variances explained by flock date and model residual error. ^3^ RMSE = root mean squared error.

## Data Availability

The original contributions presented in the study are included in the article, and further inquiries can be directed to the corresponding author.

## References

[1] (2024). Regulation (EU) 2024/1143 Regulation (EU) 2024/1143 of the European Parliament and of the Council of 11 April 2024 on Geographical Indications for Wine, Spirit Drinks and Agricultural Products, as Well as Traditional Specialities Guaranteed and Optional Quality Terms for Agricultural Products, Amending Regulations (EU) No 1308/2013, (EU) 2019/787 and (EU) 2019/1753 and Repealing Regulation (EU) No 1151/2012.

[2] FAO FAOSTAT. https://www.fao.org/faostat/en/#home.

[3] (2023). Activities Report of the PDO “Queso Manchego”.

[4] (2004). Regulation (EC) No. 853/2004 Regulation (EC) No. 853/2004 of the European Parliament and of the Council of 29 April 2004 Laying Down Specific Hygiene Rules for Food of Animal Origin.

[5] Pirisi A., Lauret A., Dubeuf J.P. (2007). Basic and Incentive Payments for Goat and Sheep Milk in Relation to Quality. Small Rumin. Res..

[6] Olde Riekerink R.G.M., Barkema H.W., Veenstra W., Berg F.E., Stryhn H., Zadoks R.N. (2007). Somatic Cell Count During and Between Milkings. J. Dairy Sci..

[7] Leitner G., Silanikove N., Merin U. (2008). Estimate of Milk and Curd Yield Loss of Sheep and Goats with Intrammamary Infection and Its Relation to Somatic Cell Count. Small Rumin. Res..

[8] Gonzalo C., Ariznabarreta A., Carriedo J.A., San Primitivo F. (2002). Mammary Pathogens and Their Relationship to Somatic Cell Count and Milk Yield Losses in Dairy Ewes. J. Dairy Sci..

[9] González-Viñas M.A., Poveda J., García Ruiz A., Cabezas L. (2001). Changes in Chemical, Sensory and Rheological Characteristics of Manchego Cheeses during Ripening. J. Sens. Stud..

[10] Raynal-Ljutovac K., Pirisi A., de Crémoux R., Gonzalo C. (2007). Somatic Cells of Goat and Sheep Milk: Analytical, Sanitary, Productive and Technological Aspects. Small Rumin. Res..

[11] Ferragina A., Cipolat-Gotet C., Cecchinato A., Pazzola M., Dettori M.L., Vacca G.M., Bittante G. (2017). Prediction and Repeatability of Milk Coagulation Properties and Curd-Firming Modeling Parameters of Ovine Milk Using Fourier-Transform Infrared Spectroscopy and Bayesian Models. J. Dairy Sci..

[12] Pazzola M., Cipolat-Gotet C., Bittante G., Cecchinato A., Dettori M.L., Vacca G.M. (2018). Phenotypic and Genetic Relationships between Indicators of the Mammary Gland Health Status and Milk Composition, Coagulation, and Curd Firming in Dairy Sheep. J. Dairy Sci..

[13] Bittante G., Penasa M., Cecchinato A. (2012). Invited Review: Genetics and Modeling of Milk Coagulation Properties. J. Dairy Sci..

[14] Garzón A., Figueroa A., Caballero-Villalobos J., Angón E., Arias R., Perea J.M. (2021). Derivation of Multivariate Indices of Milk Composition, Coagulation Properties, and Curd Yield in Manchega Dairy Sheep. J. Dairy Sci..

[15] Pazzola M. (2019). Coagulation Traits of Sheep and Goat Milk. Animals.

[16] Martí-De Olives A., Peris C., Molina M.P. (2020). Effect of Subclinical Mastitis on the Yield and Cheese-Making Properties of Ewe’s Milk. Small Rumin. Res..

[17] Albenzio M., Caroprese M., Santillo A., Marino R., Taibi L., Sevi A. (2004). Effects of Somatic Cell Count and Stage of Lactation on the Plasmin Activity and Cheese-Making Properties of Ewe Milk. J. Dairy Sci..

[18] Cecchinato A., Bittante G. (2016). Genetic and Environmental Relationships of Different Measures of Individual Cheese Yield and Curd Nutrients Recovery with Coagulation Properties of Bovine Milk. J. Dairy Sci..

[19] Pazzola M., Stocco G., Ferragina A., Bittante G., Dettori M.L., Vacca G.M., Cipolat-Gotet C. (2023). Cheese Yield and Nutrients Recovery in the Curd Predicted by Fourier-Transform Spectra from Individual Sheep Milk Samples. J. Dairy Sci..

[20] Buffa M.N., Trujillo A.J., Pavia M., Guamis B. (2001). Changes in Textural, Microstructural, and Colour Characteristics during Ripening of Cheeses Made from Raw, Pasteurized or High-Pressure-Treated Goats’ Milk. Int. Dairy J..

[21] McMahon D.J., Brown R.J. (1982). Evaluation of Formagraph for Comparing Rennet Solutions. J. Dairy Sci..

[22] Bittante G., Pellattiero E., Malchiodi F., Cipolat-Gotet C., Pazzola M., Vacca G.M., Schiavon S., Cecchinato A. (2014). Quality Traits and Modeling of Coagulation, Curd Firming, and Syneresis of Sheep Milk of Alpine Breeds Fed Diets Supplemented with Rumen-Protected Conjugated Fatty Acid. J. Dairy Sci..

[23] Ali A.K.A., Shook G.E. (1980). An Optimum Transformation for Somatic Cell Concentration in Milk. J. Dairy Sci..

[24] Legarra A., Ramón M., Ugarte E., Pérez-Guzmán M.D., Arranz J. (2007). Economic Weights of Somatic Cell Score in Dairy Sheep. Animal.

[25] Díaz Ramírez J., Bobbo T., Matera R., Gómez-Carpio M., Cimmino R., Pedota G., Biffani S., Neglia G. (2025). Genetic Parameters for Somatic Cell Score, Differential Somatic Cell Count, and Milk Electrical Conductivity in Dairy Buffaloes. Ital. J. Anim. Sci..

[26] Lopez-Fandiño R., Carrascosa A.V., Olano A. (1996). The Effects of High Pressure on Whey Protein Denaturation and Cheese-Making Properties of Raw Milk. J. Dairy Sci..

[27] Melilli C., Lynch J.M., Carpino S., Barbano D.M., Licitra G., Cappa A. (2002). An Empirical Method for Prediction of Cheese Yield. J. Dairy Sci..

[28] Othmane M.H., Carriedo J.A., de la Fuente Crespo L.F., San Primitivo F. (2002). An Individual Laboratory Cheese-Making Method for Selection in Dairy Ewes. Small Rumin. Res..

[29] Cipolat-Gotet C., Cecchinato A., De Marchi M., Bittante G. (2013). Factors Affecting Variation of Different Measures of Cheese Yield and Milk Nutrient Recovery from an Individual Model Cheese-Manufacturing Process. J. Dairy Sci..

[30] Tiberio M.L., Diniz F. (2014). Sheep and Goat Production in Portugal: A Dynamic View. Mod. Econ..

[31] Scintu M.F., Piredda G. (2007). Typicity and Biodiversity of Goat and Sheep Milk Products. Small Rumin. Res..

[32] Dubeuf J.-P., de A. Ruiz Morales F., Castel Genis J.M. (2010). Initiatives and Projects to Promote the Mediterranean Local Cheeses and Their Relations to the Development of Livestock Systems and Activities. Small Rumin. Res..

[33] Ugarte E., Legarra A., Gabiña D., Sanna S. (2003). Scientific Background of the Selection Program in the Latxa Breed. Breeding Programmes for Improving the Quality and Safety of Products: New Traits, Tools, Rules and Organization?.

[34] Figueroa A., Caballero-Villalobos J., Angón E., Arias R., Garzón A., Perea J.M. (2020). Using Multivariate Analysis to Explore the Relationships between Color, Composition, Hygienic Quality, and Coagulation of Milk from Manchega Sheep. J. Dairy Sci..

[35] Caballero-Villalobos J., Garzón A., Angón E., Arias R., Cecchinato A., Amalfitano N., Perea J.M. (2024). Exploring Breed-Specific Milk Coagulation in Spanish Dairy Sheep: A Canonical Correlation Approach. Animals.

[36] Correddu F., Gaspa G., Cesarani A., Macciotta N.P.P. (2022). Phenotypic and Genetic Characterization of the Occurrence of Noncoagulating Milk in Dairy Sheep. J. Dairy Sci..

[37] Stocco G., Cipolat-Gotet C., Summer A., Tiezzi F., Blotta S., Negro A., Castiglioni B., Biffani S. (2025). Modeling the Relationships among Technological Properties of Sheep Individual Animal Factors, Milk Composition, and Minerals Using Generalized Additive Mixed Models. J. Dairy Sci..

[38] Barillet F., Marie C., Jacquin M., Lagriffoul G., Astruc J.M. (2001). The French Lacaune Dairy Sheep Breed: Use in France and Abroad in the Last 40 Years. Livest. Prod. Sci..

[39] Vouraki S., Astruc J.-M., Lagriffoul G., Rupp R., Banos G., Arsenos G. (2025). Genotype-by-Environment Interactions and Response to Selection for Milk Production Traits in Lacaune Sheep from Greece and France. Vet. Sci..

[40] Manuelian C.L., Penasa M., Giangolini G., Boselli C., Currò S., De Marchi M. (2019). Short Communication: Fourier-Transform Mid-Infrared Spectroscopy to Predict Coagulation and Acidity Traits of Sheep Bulk Milk. J. Dairy Sci..

[41] Paschino P., Vacca G.M., Dettori M.L., Pazzola M. (2019). An Approach for the Estimation of Somatic Cells’ Effect in Sarda Sheep Milk Based on the Analysis of Milk Traits and Coagulation Properties. Small Rumin. Res..

[42] Jiménez L., Poveda Colado J.M., Garzón Sigler A.I., Martínez Marín A.L., Núñez Sánchez N., Asensio J.R., Pérez-Guzmán Palomares M.D., Arias Sánchez R. (2018). Composition and Colour Indices of Sheep’s Bulk-Tank Milk Are Influenced by Production Practices. Ital. J. Anim. Sci..

[43] Giovanetti V., Boe F., Decandia M., Bomboi G.C., Atzori A.S., Cannas A., Molle G. (2019). Milk Urea Concentration in Dairy Sheep: Accounting for Dietary Energy Concentration. Animals.

[44] Marshall A.C., Vigolo V., Marchi M.D., Lopez-Villalobos N., Loveday S.M., Weeks M., McNabb W. (2025). Effect of Protein Polymorphisms on Milk Composition, Coagulation Properties, and Protein Profile in Dairy Sheep. Int. Dairy J..

[45] Bittante G., Contiero B., Cecchinato A. (2013). Prolonged Observation and Modelling of Milk Coagulation, Curd Firming, and Syneresis. Int. Dairy J..

[46] Guinee T.P., Mulholland E.O., Kelly J., Callaghan D.J.O. (2007). Effect of Protein-to-Fat Ratio of Milk on the Composition, Manufacturing Efficiency, and Yield of Cheddar Cheese. J. Dairy Sci..

[47] Milán M.J., Caja G., González-González R., Fernández-Pérez A.M., Such X. (2011). Structure and Performance of Awassi and Assaf Dairy Sheep Farms in Northwestern Spain. J. Dairy Sci..

[48] Ikonen T., Ahlfors K., Kempe R., Ojala M., Ruottinen O. (1999). Genetic Parameters for the Milk Coagulation Properties and Prevalence of Noncoagulating Milk in Finnish Dairy Cows. J. Dairy Sci..

[49] Bencini R. (2002). Factors Affecting the Clotting Properties of Sheep Milk. J. Sci. Food Agric..

[50] Bittante G. (2011). Modeling Rennet Coagulation Time and Curd Firmness of Milk. J. Dairy Sci..

[51] García V., Rovira S., Boutoial K., López M.B. (2014). Improvements in Goat Milk Quality: A Review. Small Rumin. Res..

[52] Cipolat-Gotet C., Cecchinato A., Pazzola M., Dettori M.L., Bittante G., Vacca G.M. (2016). Potential Influence of Herd and Animal Factors on the Yield of Cheese and Recovery of Components from Sarda Sheep Milk, as Determined by a Laboratory Bench-Top Model Cheese-Making. Int. Dairy J..

[53] Vacca G.M., Stocco G., Dettori M.L., Bittante G., Pazzola M. (2020). Goat Cheese Yield and Recovery of Fat, Protein, and Total Solids in Curd Are Affected by Milk Coagulation Properties. J. Dairy Sci..

[54] Fonseca M., Kurban D., Roy J.-P., Santschi D.E., Molgat E., Yang D.A., Dufour S. (2025). Usefulness of Differential Somatic Cell Count for Udder Health Monitoring: Identifying Referential Values for Differential Somatic Cell Count in Healthy Quarters and Quarters with Subclinical Mastitis. J. Dairy Sci..

[55] Gelasakis A.I., Mavrogianni V.S., Petridis I.G., Vasileiou N.G.C., Fthenakis G.C. (2015). Mastitis in Sheep–The Last 10 Years and the Future of Research. Vet. Microbiol..

[56] Kaskous S., Farschtschi S., Pfaffl M.W. (2022). Physiological Aspects of Milk Somatic Cell Count in Small Ruminants—A Review. Dairy.

[57] De la Cruz M., Serrano E., Montoro V., Marco J., Romeo M., Baselga R., Albizu I., Amorena B. (1994). Etiology and Prevalence of Subclinical Mastitis in the Manchega Sheep at Mid-Late Lactation. Small Rumin. Res..

[58] Arias R., Oliete B., Ramón M., Arias C., Gallego R., Montoro V., Gonzalo C., Pérez-Guzmán M.D. (2012). Long-Term Study of Environmental Effects on Test-Day Somatic Cell Count and Milk Yield in Manchega Sheep. Small Rumin. Res..

[59] Nudda A., Feligini M., Battacone G., Macciotta N.P.P., Pulina G. (2003). Effects of Lactation Stage, Parity, β-Lactoglobulin Genotype and Milk SCC on Whey Protein Composition in Sarda Dairy Ewes. Ital. J. Anim. Sci..

[60] Giadinis N.D., Arsenos G., Tsakos P., Psychas V., Dovas C.I., Papadopoulos E., Karatzias H., Fthenakis G.C. (2012). “Milk-Drop Syndrome of Ewes”: Investigation of the Causes in Dairy Sheep in Greece. Small Rumin. Res..

[61] Desidera F., Skeie S.B., Devold T.G., Inglingstad R.A., Porcellato D. (2025). Fluctuations in Somatic Cell Count and Their Impact on Individual Goat Milk Quality throughout Lactation. J. Dairy Sci..

[62] Danieli B., Schogor A.L.B., Zucchi J., Neto A.T. (2025). Cows with High SCC Exhibit Poorer Performance and Milk Quality, Regardless of the Season. Dairy.

[63] (2019). Regulation EU 2019/6 Regulation (EU) 2019/6 of the European Parliament and of the Council of 11 December 2018 on Veterinary Medicinal Products and Repealing Directive 2001/82/EC.

[64] (2024). ESROM Memoria de Actividades.

[65] Vrcan M., Suárez-Vega A., Marina H., Dzidic A., Arranz J.J., Gutiérrez-Gil B. (2026). Genome-Wide Analyses to Identify Genomic Regions Associated with Udder Morphology Traits in Dairy Sheep. J. Dairy Sci..

[66] Battacone G., Cannas E.A., Mazzette A., Dimauro C., Enne G. (2005). Why Does the Increase of Plasmin Worsen the Coagulation Properties of Milk in Dairy Sheep?. Ital. J. Anim. Sci..

[67] Nudda A., Carta S., Battacone G., Pulina G. (2023). Feeding and Nutritional Factors That Affect Somatic Cell Counts in Milk of Sheep and Goats. Vet. Sci..

[68] Bobbo T., Cipolat-Gotet C., Bittante G., Cecchinato A. (2016). The Nonlinear Effect of Somatic Cell Count on Milk Composition, Coagulation Properties, Curd Firmness Modeling, Cheese Yield, and Curd Nutrient Recovery. J. Dairy Sci..

[69] Antanaitis R., Juozaitienė V., Jonike V., Baumgartner W., Paulauskas A. (2021). Milk Lactose as a Biomarker of Subclinical Mastitis in Dairy Cows. Animals.

[70] Valoppi D., Stocco G., Summer A., Niero G., Penasa M., Ablondi M., Cipolat-Gotet C. (2025). Exploring the Effect of Total and Differential Somatic Cell Traits on the Mineral Profile of Milk from Individual Dairy Cows. J. Dairy Sci..

[71] Ariton A.M., Neculai-Văleanu A.S., Poroșnicu I. (2024). The Impact of Increased Somatic Cell Count on Cow Milk Acidity and Lactose Content. Sci. Papers. Ser. D. Anim. Sci..

[72] Abdel-Salam Z., Attala S.A., Daoud E., Harith M.A. (2015). Monitoring of Somatic Cells in Milk via Laser Analytical Techniques for the Early Detection of Mastitis. Dairy Sci. Technol..

[73] Viguier C., Arora S., Gilmartin N., Welbeck K., O’Kennedy R. (2009). Mastitis Detection: Current Trends and Future Perspectives. Trends Biotechnol..

[74] Scarso S., McParland S., Visentin G., Berry D.P., McDermott A., Marchi M. (2017). Genetic and Nongenetic Factors Associated with Milk Color in Dairy Cows. J. Dairy Sci..

[75] Chudy S., Bilska A., Kowalski R., Teichert J. (2020). Colour of Milk and Milk Products in CIE L*a*b* Space. Med. Weter..

[76] Garzón A., Perea J.M., Angón E., Ryan E.G., Keane O.M., Caballero-Villalobos J. (2024). Exploring Interrelationships between Colour, Composition, and Coagulation Traits of Milk from Cows, Goats, and Sheep. Foods.

[77] Jaeggi J.J., Govindasamy-Lucey S., Berger Y.M., Johnson M.E., McKusick B.C., Thomas D.L., Wendorff W.L. (2003). Hard Ewe’s Milk Cheese Manufactured from Milk of Three Different Groups of Somatic Cell Counts. J. Dairy Sci..

[78] Bianchi L., Bolla A., Budelli E., Caroli A., Casoli C., Pauselli M., Duranti E. (2004). Effect of Udder Health Status and Lactation Phase on the Characteristics of Sardinian Ewe Milk. J. Dairy Sci..

[79] Leitner G., Chaffer M., Shamay A., Shapiro F., Merin U., Ezra E., Saran A., Silanikove N. (2004). Changes in Milk Composition as Affected by Subclinical Mastitis in Sheep. J. Dairy Sci..

[80] Lianou D.T., Michael C.K., Gougoulis D.A., Cripps P.J., Vasileiou N.G.C., Solomakos N., Petinaki E., Katsafadou A.I., Angelidou E., Arsenopoulos K.V. (2022). High Milk Somatic Cell Counts and Increased Teladorsagia Burdens Overshadow Non-Infection-Related Factors as Predictors of Fat and Protein Content of Bulk-Tank Raw Milk in Sheep and Goat Farms. Foods.

[81] Vivar-Quintana A.M., Beneitez De La Mano E., Revilla I. (2006). Relationship between Somatic Cell Counts and the Properties of Yoghurt Made from Ewes’ Milk. Int. Dairy J..

[82] Tvarožková K., Tančin V., Uhrinčať M., Mačuhová L., Mikláš Š. (2021). The Impact of Somatic Cell Count on Milk Yield and Composition. Acta Fytotech. Zootech..

[83] Pirisi A., Piredda G., Papoff C., di Salvo R., Pintus S., Garro G. Influence of Somatic Cell Count on Ewe’s Milk Composition, Cheese Yield and Cheese Quality. Proceedings of the 6th Great Lake Dairy Sheep Symposium.

[84] Kelly A.L., O’Flaherty F., Fox P.F. (2006). Indigenous Proteolytic Enzymes in Milk: A Brief Overview of the Present State of Knowledge. Int. Dairy J..

[85] Poulsen N.A., Buitenhuis A.J., Larsen L.B. (2015). Phenotypic and Genetic Associations of Milk Traits with Milk Coagulation Properties. J. Dairy Sci..

[86] Albenzio M., Santillo A., Kelly A.L., Caroprese M., Marino R., Sevi A. (2015). Activities of Indigenous Proteolytic Enzymes in Caprine Milk of Different Somatic Cell Counts. J. Dairy Sci..

[87] Ahmadi E., Markoska T., Huppertz T., Vasiljevic T. (2024). Structural Properties of Casein Micelles with Adjusted Micellar Calcium Phosphate Content. Foods.

[88] Pellegrini O., Remeuf F., Rivemale M. (1994). Evolution Des Caracteristiques Physico-Chimiques et Des Parametres de Coagulation Du Lait de Brebis Collecte Dans La Region de Roquefort. Lait.

[89] Maristela R., Natalia R., Gerardo C., Jordi S., Gabriel L. (2015). Effect of Subclinical Intrammamay Infection on Milk Quality in Dairy Sheep: I. Fresh-Soft Cheese Produced from Milk of Uninfected and Infected Glands and from Their Blends. Small Rumin. Res..

[90] Wellnitz O., Bruckmaier R.M. (2012). The Innate Immune Response of the Bovine Mammary Gland to Bacterial Infection. Vet. J..

[91] Suriyasathaporn W., Schukken Y.H., Nielen M., Brand A. (2000). Low Somatic Cell Count: A Risk Factor for Subsequent Clinical Mastitis in a Dairy Herd. J. Dairy Sci..

[92] Ikonen T., Morri S., Tyrisevä A.-M., Ruottinen O., Ojala M. (2004). Genetic and Phenotypic Correlations Between Milk Coagulation Properties, Milk Production Traits, Somatic Cell Count, Casein Content, and PH of Milk. J. Dairy Sci..

[93] González-Rodríguez M.C., Gonzalo C., San Primitivo F., Cármenes P. (1995). Relationship Between Somatic Cell Count and Lntramammary Infection of the Half Udder in Dairy Ewes. J. Dairy Sci..

[94] Mazal G., Vianna P.C.B., Santos M.V., Gigante M.L. (2007). Effect of Somatic Cell Count on Prato Cheese Composition. J. Dairy Sci..

[95] Vianna P.C.B., Mazal G., Santos M.V., Bolini H.M.A., Gigante M.L. (2008). Microbial and Sensory Changes throughout the Ripening of Prato Cheese Made from Milk with Different Levels of Somatic Cells. J. Dairy Sci..

[96] Revilla I., Rodríguez-Nogales J.M., Vivar-Quintana A.M. (2011). Effects of Somatic Cells on the Protein Profile of Hard Ovine Cheese Produced from Different Breeds. J. Dairy Res..

[97] Garzón A., Perea J.M., Arias R., Angón E., Caballero-Villalobos J. (2023). Efficiency of Manchega Sheep Milk Intended for Cheesemaking and Determination of Factors Causing Inefficiency. Animals.

[98] Summer A., Franceschi P., Formaggioni P., Malacarne M. (2015). Influence of Milk Somatic Cell Content on Parmigiano-Reggiano Cheese Yield. J. Dairy Res..

[99] Libera K., Konieczny K., Grabska J., Smulski S., Szczerbal I., Szumacher-Strabel M., Pomorska-Mól M. (2021). Potential Novel Biomarkers for Mastitis Diagnosis in Sheep. Animals.

[100] Świderek W.P., Charon K.M., Winnicka A., Gruszczyńska J., Pierzchała M. (2016). Physiological Threshold of Somatic Cell Count in Milk of Polish Heath Sheep and Polish Lowland Sheep. Ann. Anim. Sci..

[101] Chambers G., Lawrence K., Grinberg A., Velathanthiri N., Ridler A., Laven R. (2026). Subclinical Mastitis in New Zealand Grazing Dairy Ewes 2: Relationships among Somatic Cell Count, California Mastitis Test, and Milk Culture, and Risk Factors for Elevated Aerobic Plate Count. J. Dairy Sci..

